# Draft Genome Sequence of Immunobiotic *Lactobacillus rhamnosus* Strain IBL027, a Potential Adjuvant for Mucosal Vaccine Development

**DOI:** 10.1128/genomeA.01268-17

**Published:** 2017-12-14

**Authors:** María Guadalupe Vizoso-Pinto, Lucila Saavedra, Elvira Maria Hebert, Fernanda Raya Tonetti, Leonardo Albarracín, Susana Alvarez, Haruki Kitazawa, Julio Villena

**Affiliations:** a Infection Biology Laboratory, INSIBIO (UNT-CONICET), Faculty of Medicine of the National University of Tucumán, Tucumán, Argentina; b Reference Centre for Lactobacilli (CERELA-CONICET), San Miguel de Tucumán, Tucumán, Argentina; c Food and Feed Immunology Group, Laboratory of Animal Products Chemistry, Graduate School of Agricultural Science, Tohoku University, Sendai, Japan; d Livestock Immunology Unit, International Education and Research Center for Food Agricultural Immunology (CFAI), Graduate School of Agricultural Science, Tohoku University, Sendai, Japan

## Abstract

The genome sequence of the immunomodulatory strain *Lactobacillus rhamnosus* strain IBL027 is described here. The reads were assembled into contigs with a total size 2,898,501 bp. The genome information will be useful for further specific genetic studies of this strain to evaluate its immunomodulatory and biotechnological properties as a vaccine adjuvant.

## GENOME ANNOUNCEMENT

*Lactobacillus rhamnosus* strain IBL027 was originally isolated from a vaginal swab from a healthy woman. Preliminary studies in mouse models have demonstrated that this strain has immunomodulatory activities (M. P. Raimondo, M. F. Raya-Tonetti, S. Salva, S. Alvarez, J. Villena, M. G. Vizoso-Pinto, presented at V International Symposium on Lactic Acid Bacteria: Benefiting from Lactic Acid Bacteria—Progress in Health and Food, Tucumán, Argentina, 19 to 21 October 2016; L. Arce, M. F. Raya Tonetti, M. Müller, J. Villena, M. G. Vizoso Pinto, presented at XII Argentine Congress of Virology, Buenos Aires, Argentina, 26 to 28 September 2017). The adjuvant capacity of *L. rhamnosus* strain IBL027 was evaluated using oral commercial vaccines for rotavirus. *L. rhamnosus* strain IBL027, when used as an adjuvant for oral immunization of mice with rotavirus vaccine, significantly improved humoral and cellular immune responses at the mucosal and systemic compartments. The IBL027 strain was able to increase the levels of specific IgA in intestinal fluid and the production of gamma interferon (IFN-γ) by lymphocytes from spleen and Payer’s patches (Raimondo et al, unpublished). It was also found that the immunomodulatory bacterium-like particles (IBLP) obtained by acid heat treatment of *L. rhamnosus* strain IBL027 act as mucosal adjuvants by enhancing intestinal immunity when administered with experimental vaccine directed against hepatitis E virus (Arce et al, unpublished). These results show that *L. rhamnosus* strain IBL027 may be suitable for enhancing the effectiveness of commercial vaccines and for the development of new vaccine formulations.

Here, we present a draft genome sequence of *L. rhamnosus* strain IBL027 that was sequenced using a whole-genome shotgun (WGS) strategy on an Illumina MiSeq sequencer. Paired reads with lengths of 300 bp were obtained, corresponding to 150-fold coverage. Quality-filtered reads were assembled using NGen (version 12.2.0; DNAStar). The RAST server and the NCBI’s Prokaryotic Genome Annotation Pipeline were used for the functional annotation of predicted genes (1). tRNAs and rRNAs were identified by tRNAscan-SE and RNAmmer, respectively (2, 3).

The draft genome of *L. rhamnosus* strain IBL027 consists of 2,898,501 bp, with a mean G+C content of 46.8%. A total of 2,860 coding sequences (CDS), 56 structural tRNAs, and 4 rRNAs were predicted. Among all CDS, 2,725 were assigned to known protein functions, while 135 remained as hypothetical proteins. Additionally, there are 170 RAST subsystems represented in the genome, which represent only 39% of the assigned sequences.

Genomic analysis using BAGEL3 (4), RAST (1), and BLAST demonstrated the presence of a putative bacteriocin class II cluster in contig_38 and putative fibronectin-binding protein in contig_7. Similar to other strains of the species, *L. rhamnosus* strain IBL027 contains genes responsible for exopolysaccharide biosynthesis. No remarkable antibiotic resistance or virulence-associated genes were found.

The genome information of *L. rhamnosus* strain IBL027 presented here will be useful for further studies of the specific genetic features of this strain and for its biotechnological application in the development of novel mucosal vaccines. In addition, the genome information will be of value for understanding the mechanisms of its immunomodulatory properties.

### Accession number(s).

This whole-genome shotgun project has been deposited at DDBJ/EMBL/GenBank under the accession no. NBVL00000000. The version described in this paper is version NBVL01000000.
